# Current Clinical Trials in Pemphigus and Pemphigoid

**DOI:** 10.3389/fimmu.2019.00978

**Published:** 2019-05-03

**Authors:** Kentaro Izumi, Katja Bieber, Ralf J. Ludwig

**Affiliations:** ^1^Department of Dermatology, Hokkaido University Graduate School of Medicine, Sapporo, Japan; ^2^Lübeck Institute of Experimental Dermatology, Center for Research on Inflammation of the Skin, University of Lübeck, Lübeck, Germany

**Keywords:** autoimmune blistering skin disease, pemphigus, pemphigoid, clinical trial, treatment

## Abstract

Autoimmune bullous dermatoses (AIBDs) are a group of rare chronic inflammatory skin diseases, which clinically manifest as blisters and erosions of the skin and/or mucosa. Immunologically, AIBDs are characterized and caused by autoantibodies targeting adhesion molecules in the skin and mucosa. According to the histological location of the blistering, AIBDs are classified into the following two main subtypes: pemphigus (intraepidermal blistering) and pemphigoid (subepidermal blistering). Most AIBDs were potentially life-threatening diseases before the advent of immunosuppressive drugs, especially systemic steroid therapies, which suppress pathogenic immunological activity. Although there have been recent advancements in the understanding of the pathogenesis of AIBDs, glucocorticosteroids and/or adjuvant immunosuppressive drugs are still needed to control disease activity. However, the long-term use of systemic immunosuppression is associated with major adverse events, including death. Based on the growing understanding of AIBD pathogenesis, novel treatment targets have emerged, some of which are currently being evaluated in clinical trials. Within this article, we review the current clinical trials involving pemphigus and pemphigoid and discuss the rationale that lead to these trials. Overall, we aim to foster insights into translational research in AIBDs to improve patient care.

## Introduction

Autoimmune bullous dermatoses (AIBDs) are a heterogeneous group of skin diseases that are characterized and caused by autoantibodies targeting adhesion molecules in the skin and/or mucous membranes. Depending on the targeted adhesion molecules and the location of the blistering, AIBDs are classified into the following two major types: pemphigus diseases with autoantibodies targeting desmosomal proteins (1) and pemphigoid diseases with autoantibodies targeting the structural proteins of dermal-epidermal junction (2). In pemphigus diseases, including pemphigus vulgaris (PV) and pemphigus foliaceus (PF), autoantibody binding leads to the disruption of epidermal adhesion, resulting in the clinical finding of flaccid blisters and the histological finding of intraepidermal blistering (1). In pemphigoid diseases, the linear deposition of autoantibodies along the dermal-epidermal junction causes subepidermal blistering, resulting in tense blisters. Based on the target molecules of the autoantibodies and the clinical manifestations, pemphigoid diseases are classified as bullous pemphigoid (BP), mucous membrane pemphigoid (MMP), epidermolysis bullosa acquisita (EBA), anti-laminin-γ1/p200 pemphigoid (p200), pemphigoid gestationis (PG), lichen planus pemphigoides (LPP), linear IgA bullous dermatosis (LAD), and dermatitis herpetiformis (DH), which is associated with gluten-sensitive enteropathy and characteristic granular IgA deposits in the upper dermis (3–5).

Although the advent of systemic steroid therapy significantly improved the prognosis of AIBDs, these groups of diseases are still potentially life-threatening, now mainly due to adverse events resulting from corticosteroid treatment (6). Due to the chronicity of AIBDs, the prolonged administration of systemic steroids is often needed to induce and maintain clinical remission, leading to various adverse effects such as cytopenia, diabetes mellitus, osteoporosis, hypertension, gastrointestinal ulcers, and infections due to immunosuppression (7). Furthermore, severe infection induced by an immunocompromised state is one of the most important causes of death during AIBD treatment (8, 9). Thus, the development of alternative treatment modalities that have fewer adverse events is urgently needed for the treatment of AIBD patients.

**Figure 1:**
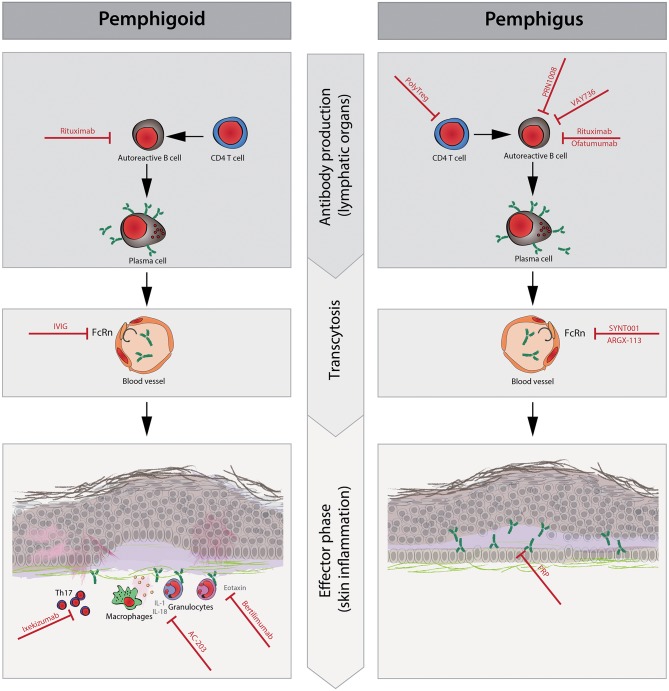
Schematic representation of the pathophysiology and new therapeutic targets of pemphigus and pemphigoid diseases. The pathophysiology of pemphigus and pemphigoid diseases consists of the following three phases: (1) CD4+T cells promote autoreactive B cell activation, proliferation, and differentiation to plasma cells that produce pathogenic autoantibodies. (2) Circulating pathogenic antibodies are transferred to the dermal epidermal junction or intracellular space of the epidermis. Neonatal Fc receptor (FcRn) plays a role in prolonging the half-life of IgG antibodies during this phase. (3) After the binding of pathogenic autoantibodies to target molecules, pro-inflammatory cells such as granulocytes and macrophages are recruited to the immune complex in lesional skin by chemokines (e.g., eotaxin). Then, granulocytes elicit reactive oxygen species (ROS), elastases, and proteases, resulting in tissue damage such as blisters and/or erythema, which are clinical symptoms in pemphigoid diseases but not in pemphigus diseases. Cytokines [e.g., interleukin (IL)-1beta and IL-18] and Th17 polarization are thought to enhance local inflammation. During the antibody production phase, rituximab and ofatumumab deplete autoreactive B cells to prevent their differentiation to plasma cells. PolyTregs act on CD4+ T cells, and VAY736 and PRN1008 act on autoreactive B cells, resulting in less activation of autoreactive B cells. In the transcytosis phase, intravenous immunoglobulin (IVIg), SYNT001, and ARGX-113 saturate FcRn, contributing to the shortened half-life of pathogenic autoantibodies. In the effector phase, ixekizumab restores Th17 polarization and suppresses inflammatory augmentation. AC-203 modulates cytokines such as IL-1beta and IL-18, contributing to decreased inflammation. The inhibition of eotaxin with bertilimumab ameliorates the recruitment of eosinophils to local inflammation sites in pemphigoid disease, especially bullous pemphigoid. Platelet-rich plasma (PRP) is thought to promote wound healing in erosions.

Based on the growing understanding of AIBD pathogenesis (1, 10, 11), novel therapeutic targets and/or treatment modalities have been identified (12–15). Some of those new treatments are currently being evaluated in clinical trials. To foster translational AIBD research, in this article, we summarize the current clinical trials involving pemphigus and pemphigoid diseases. For this purpose, we searched clinicaltrials.gov^1^ and the EU clinical trials register^2^ through December 2018 and selected clinical trials on pemphigus or pemphigoid disease with the status “recruiting (the study is currently recruiting participants),” “active, not recruiting (the study is ongoing, and participants are receiving an intervention or being examined, but not currently being recruited or enrolled),” and “completed (the study has ended normally, and participants are no longer being examined or treated),” and an initiation in 2013 or later (Tables 1, 2, Figure 1). To provide a more comprehensive overview, we list the trials before this time frame in Supplementary Tables 1, 2. In addition, we included one clinical trial that is registered with the Australia and New Zealand Clinical Trials Registry, which was recently presented at the 5th International Pemphigus and Pemphigoid Foundation Scientific Conference in Orlando (16).

**Table 1:** Current clinical trials in pemphigus.

**NCT number**	**Disease**	**Interventions**	**Target**	**Allocation**	**Masking**	**Phase**	**Status**
NCT01930175	PV	VAY736	BAFF-R	Randomized	Double blind	2	Active, not recruiting
NCT01920477	PV	Ofatumumab	CD20	Randomized	Double blind	3	Completed
NCT03334058	PV	ARGX-113	FcRn	Single group	None	2	Recruiting
NCT02704429	PV or PF	PRN1008	BTK	Single group	None	2	Recruiting
NCT03762265	PV or PF	PRN1008	BTK	Randomized	Quadruple blind	3	Recruiting
NCT02383589	PV	Rituximab MMF	CD20 IMPDH	Randomized	Double blind	3	Active, not recruiting
NCT03239470	PV or PF	Poly Tregs	Immune tolerance	Non- randomized	None	1	Recruiting
NCT02828163	PV	PRP	Wound healing	Randomized	Double blind	3	Recruiting
NCT00784589	PV or PF	Rituximab	CD20	Randomized	None	3	Completed
NCT03075904	PV or PF	SYNT001	FcRn	Non-randomized	None	1/2	Completed

*PV, pemphigus vulgaris; PF, pemphigus foliaceus; BAFF-R, B cell activating factor of the tumor necrosis factor family receptor; FcRn, neonatal Fc receptor; BTK, Bruton's tyrosine kinase; MMF, mycophenolate mofetil; IMPDH, inosine 5′-monophosphate dehydrogenase; Tregs, regulatory T cells; PRP, Platelet rich plasma*.

**Table 2:** Current clinical trials in bullous pemphigoid.

**NCT number**	**Interventions**	**Target**	**Allocation**	**Masking**	**Phase**	**Status**
NCT03099538	Ixekizumab	IL-17	Single group	None	2	Recruiting
NCT01408550	NPB-1	FcRn	Randomized	Double blind	3	Completed
NCT03286582	AC-203	Inflammasome	Randomized	None	2	Recruiting
NCT02226146	Bertilimumab	eotaxin	Single group	None	2	Completed
NCT00525616	Rituximab	CD20	Single group	None	3	Completed

*No clinical trials are being performed for any other pemphigoid diseases. FcRn, neonatal Fc receptor*.

## Pemphigus

### Anti-CD20 in Pemphigus (Rituximab or Ofatumumab)

Rituximab is a human chimeric IgG1 monoclonal antibody targeting CD20, which is a cell surface marker expressed by B cells (17). Rituximab exerts its treatment effects via the depletion of B-cells following its binding to CD20. Regarding the treatment of pemphigus with rituximab, case reports have suggested the efficacy of rituximab as a second- or third-line therapy (18–20). There are two main rituximab regimens for pemphigus; one regimen, which is based on the lymphoma protocol, is composed of a total of 4 doses of 375 mg/m^2^ weekly infusions (21), while the other regimen, which is based on the rheumatoid arthritis protocol, consists of a total of two doses of 1,000 mg (or 500 mg) biweekly intravenous infusions (22). Prior to evaluating the efficacy of a single cycle regimen of rituximab in refractory pemphigus cases, a multicenter, single arm, phase 2/3 clinical trial was conducted in France (23). At 3 months after rituximab treatment, 18 (86%) of 21 cases achieved complete remission (CR). Furthermore, 18 (86%) of 21 cases maintained CR after a median follow-up of 34 months. In addition, 8 of these 18 cases did not receive systemic corticosteroid therapy. To evaluate the safety and efficacy of rituximab in a controlled clinical trial, a prospective, parallel-group, open-label, randomized, phase 3 clinical trial of rituximab as a first-line treatment for moderate to severe cases of pemphigus (**NCT00784589**) was initiated (Table 1). Patients with PV or PF were randomized to receive either oral prednisolone at 1.0 to 1.5 mg/kg/day that was tapered over 12–18 months or oral prednisolone at 0.5–1.0 mg/kg/day that was tapered over 3–6 months plus rituximab 1 g on days 0 and 14 and 0.5 g at months 12 and 18. The primary endpoint was the proportion of patients who achieved CR off-therapy at month 24. The results of this clinical trial were recently reported (24). Regarding the primary endpoint, 34% of patients in the prednisolone arm achieved CR at month 24. In the rituximab plus prednisolone arm, CR was reached by 89% of the patients; the difference was significant. Furthermore, fewer grade 3–4 adverse events were observed in the rituximab plus prednisone group than in the prednisone alone group (27 events in 16 out of 46 patients; mean 0.59 [SD 1.15] vs. 53 events in 29 out of 44 patients; mean 1.20 [1.25]). Overall, this trial demonstrated that the first-line treatment of pemphigus with rituximab and lower doses of prednisolone is more effective and safer compared to high-dose prednisolone treatment. Based on the results of this clinical trial, the Food and Drug Administration approved the expansion of health insurance coverage of rituximab for pemphigus vulgaris in the United States.

In addition to rituximab, ofatumumab, a fully human anti-CD20 monoclonal antibody, has been demonstrated to be safe and effective for the treatment of autoimmune disorders other than pemphigus or pemphigoid (25). Preclinical studies suggested that ofatumumab shows a high affinity for CD20 and activates complement-dependent cytotoxicity (26). A double-blind, randomized, placebo-controlled, phase 3 clinical trial evaluating the efficacy of ofatumumab in pemphigus was completed in January 2018 (**NCT01920477**). This clinical trial enrolled moderate to severe PV patients with a history of at least failure of tapered steroid therapy. Participants received a stable dose of prednisone/prednisolone from a minimum of 20 mg/day up to a maximum of 120 mg/day or 1.5 mg/kg/day for 2 weeks prior to randomization. Thirty-five patients with PV were randomized to receive either ofatumumab or a placebo. Ofatumumab at 40 mg was subcutaneously injected at week 0 and week 4. From week 8, subjects were subcutaneously administered ofatumumab 20 mg every 4 weeks through week 56. The primary endpoints were the time to sustained CR on minimal steroid therapy (prednisone/prednisolone dose to < 10 mg/day) and the duration of CR on minimal steroid therapy. The results of this study have thus far not been reported.

Similar to these two trials, another double-blind, randomized, phase 3 clinical trial of adjuvant rituximab vs. mycophenolate mofetil (MMF) as a therapy for pemphigus is ongoing (**NCT02383589**). In that study, 135 PV patients who are receiving standard systemic steroid treatment (oral prednisone 60–120 mg/day or equivalent) will randomly receive either (i) MMF and a rituximab-matched placebo or (ii) an MMF-matched placebo and rituximab. MMF is orally administered twice daily from day 1 to week 56. The initial dose of MMF is 500 mg, and the dose will be titrated to achieve a goal of 1,000 mg. Rituximab is administered at a dose of 1,000 mg intravenously on days 1, 15, 168, and 182. The primary endpoint is the proportion of patients who achieve sustained CR.

### VAY736 in Pemphigus

B cell activating factor of the tumor necrosis factor family (BAFF) is a crucial cytokine for regulating B cell development in mice and humans (27, 28). BAFF functions by binding to the BAFF receptor (BAFF-R), B cell maturation antigen, and tumor necrosis factor receptor superfamily member (29). Although at physiological concentrations BAFF cannot rescue B cell apoptosis due to a strong B cell death signal, which is transduced via the B cell receptor (BCR) stimulated by autoantigens, at higher concentrations, BAFF causes the survival of autoreactive B cells, which contributes to the pathogenesis of autoimmune diseases (27, 30, 31). For example, elevated BAFF serum levels have been detected in various autoimmune diseases such as RA, systemic lupus erythematosus, Sjögren's syndrome, and systemic sclerosis (32–34). Taken together, BAFF is a likely therapeutic target for the treatment of autoimmune diseases. Regarding clinical translation, the anti-BAFF antibody belimumab was licensed for the treatment of systemic lupus erythematosus in 2011 (35). However, clinical trials of belimumab have not been conducted in pemphigus thus far. Alternatively, with regard to the inhibition of BAFF, its receptor can be blocked to achieve similar results. BAFF-R signaling drives B cell differentiation, proliferation and survival (36). VAY736 is a novel, defucosylated, human IgG1 monoclonal antibody targeting BAFF-R, providing both enhanced antibody-dependent cellular cytotoxicity-mediated depletion of B cells and the blockade of BAFF. To investigate the safety, tolerability and efficacy of VAY736 in PV, a randomized, placebo-controlled, double-blind, phase 2 clinical trial is currently ongoing (**NCT01930175**). In this trial, 16 mild-moderate PV patients will randomly receive either intravenous VAY736 or a placebo once. The primary endpoint is the efficacy of single cycle VAY736 administration in reducing Pemphigus Disease Area Index (PDAI) scores at week 12 compared to at the baseline. No results of this trial are currently available.

### Anti-neonatal Fc Receptor (FcRn) in Pemphigus (SYNT001, ARGX-113)

FcRn plays an essential role in regulating host circulating IgG levels (37). FcRn protects IgG from intracellular digestion, leading to the prolongation of its half-life. This “IgG recycling system” is essential for host defense. However, it also maintains the concentration of circulating pathogenic IgG in various autoimmune diseases, including AIBD. Specifically, FcRn-deficient mice do not (or to a lesser extent) develop experimental AIBD after the injection of AIBD-inducing antibodies (10). Interestingly, saturation of the FcRn by the administration of high-dose human IgG (IVIg) reduces the pathogenic effects in antibody-transfer AIBD models. Thus, FcRn inhibition is one possible mode of action of IVIg therapy in AIBD (38, 39). Based on these observations, anti-FcRn targeting treatments have been developed. Currently, the following 2 clinical trials targeting FcRn are being conducted in pemphigus: SYNT001 (**NCT03075904**) and ARGX-113 (**NCT03334058**). SYNT001 is a humanized, deimmunized IgG4 monoclonal antibody that blocks the binding of FcRn to the Fc portion of IgG (40). ARGX-113 is a human IgG1-derived Fc-modified fragment with increased affinity for FcRn that reduces the circulating IgG concentration (41). In addition, both SYNT001 and ARGX-113 do not alter serum levels of albumin. As both trials are ongoing, no results have been published so far. Regarding **NCT03075904**, 16 PV or PF patients will be sequentially assigned to receive three different doses of intravenous SYNT001 weekly for either 5 or 14 weeks, and the primary endpoint is the count and percentage of adverse events (time frame; days 0–112 or days 0–175). At the 2018 pre-International Investigative Dermatology meeting in Orlando, the first results were reported for SYNT001. The infusion of SYNT001 in human subjects resulted in a rapid lowering of the circulating levels of IgG (mean total IgG reduction of 56% by day 30) with good safety and tolerability. Furthermore, 5 of the 7 subjects showed a reduction in disease activity by day 42 (42). Regarding **NCT03334058**, 12 newly diagnosed or relapsed PV patients will receive ARGX-113 intravenously. The primary endpoints are safety and tolerability up to 17 weeks.

### PRN1008 in Pemphigus

B cell receptor signaling is a key player in B cell development and function. Bruton's tyrosine kinase (BTK) belongs to the Tec family of nonreceptor tyrosine kinases, and it is a vital component of B cell receptor signaling (43). BTK is predominantly expressed by B-lymphocytes from the pre-B cell stage to the mature B cell stage (44, 45). Based on the crucial role of BTK in B cell function, it has been identified as a potential target for the treatment of autoimmune disorders. Several studies have shown that ibrutinib, which is one of the BTK inhibitors under development, binds to BTK with high affinity, leading to the inhibition of B cell receptor signaling and resulting in the reduction of B cell activation involved in autoimmunity (46). In pemphigus treatment, a previous case report showed that ibrutinib improved clinical cutaneous lesions of paraneoplastic pemphigus complicated with chronic lymphocytic leukemia (47). However, there have been no case reports or series investigating the effects of this BTK inhibitor in PNP, PV and/or PF. PRN1008 is another BTK inhibitor that was evaluated in a phase 1 clinical trial that enrolled 80 healthy volunteers (Australian New Zealand Clinical Trials Registry No. **ACTRN12614000359639**). In that study, PRN1008 was considered safe. Regarding the pharmacokinetics and pharmacodynamics, BTK occupancy of more than 90% was observed within 4 h after dosing in both the single and multiple dose regimens and was closely correlated with the maximum plasma concentration (48). Based on the promising results from the phase 1 trial, an open-label, single-armed, phase 2 clinical trial of PRN1008 for PV treatment has been conducted (**NCT02704429**). In this trial, 27 pemphigus patients (including PV and PF) received PRN1008 orally for 12 weeks with a 12-week follow-up period. The primary efficacy endpoint was the initial control of disease activity during the first 4 weeks of therapy, during which new lesions cease to form and existing lesions begin to heal, without the need for prednisone-equivalent corticosteroid doses >0.5 mg/kg/day. More than 50% of patients have achieved control of disease activity within 4 weeks of starting PRN1008 thus far^3^. Furthermore, Principia has recently initiated a global, randomized, double-blind, placebo-controlled, pivotal phase 3 clinical trial (**NCT03762265**) in approximately 120 PV or PF patients to evaluate PRN1008 vs. a placebo. The primary endpoint is the proportion of participants who are in CR from week ≤ 29 to 37 with a prednisone dose of ≤ 5 mg/day.

### Polyclonal Regulatory T Cells (PolyTregs) in Pemphigus

Among the T cell subtypes, regulatory T cells (Tregs) play an important role in regulating the immune system and preventing autoimmune disease development. Based on findings in animal models, including AIBD (49, 50), it is hypothesized that naturally occurring Tregs may be utilized for the treatment of autoimmune diseases and potentially replace the use of chronic immunosuppressive therapies that are associated with multiple adverse effects. A clinical trial of Treg adoptive therapy was started by treating graft vs. host diseases (GVHD) with expanded allogeneic Tregs (51). There has been a small study demonstrating the safe administration of autologous Tregs with decreased disease activity in patients with insulin-dependent diabetes (52). Subsequently, Brunstein et al. reported that HLA-matched umbilical cord blood-derived Tregs decreased the incidence of GVHD after double umbilical cord blood transplantation (53), indicating the potential efficacy and safety profile of the passive transfer of autologous Tregs in humans. The application of Tregs for lupus, cancer and organ transplantation has been addressed (54). The suppressive effects of Tregs have been reported by studies using an active PV mouse model (55). A recent study indicated that Treg induction via the anti-CD28 antibody reduces pathogenic IgG directing desmoglein (Dsg) 3 in the HLA-DRB1^*^04:02- transgenic PV mouse model (56). Although Tregs are expected to improve PV symptoms, there are no case reports or case series of autologous Treg injections in patients with PV to date. To evaluate the effects of Tregs on the manifestation of PV, a nonrandomized, open-label, phase 1 clinical trial is ongoing (**NCT03239470**). In this trial, 12 PV or PF patients will receive one infusion of autologous expanded Tregs (CD4+CD127lo/negCD25+) at one of the following doses: either 2.5 x 10^8^ poly Tregs or 10 × 10^8^ poly Tregs. The primary endpoint is the number of significant grade 3 or higher adverse events by week 52. The results from this study have thus far not been reported.

### Comparison of Injections of Steroids to Autologous Platelet-Rich Plasma (PRP) in Oral Erosions in PV

Oral erosive lesions are a major hallmark of PV. These oral lesions cause severe pain, resulting in problems with eating and drinking (57). To improve oral lesions in PV, adjuvant topical or intralesional steroids are used, and based on evidence from case reports, the treatment is effective (58, 59). To date, however, no controlled clinical trial of the treatment of oral lesions in PV has been performed. PRP, which is concentrated plasma derived from autologous whole blood, is believed to promote wound healing (60). In a case report series, El-Komy et al. reported that six of seven PV patients showed improvement of their oral PDAI scores after PRP intralesional injection (61). To evaluate the effects of PRP in PV, an open-label, dose-escalation, multicenter phase 1 trial using autologous PRP has been conducted in adults with active PV (**NCT02828163**). This clinical trial was designed to compare the effects of PRP to those of intralesional steroid injection. Eleven PV patients received a 10 mg/mL triamcinolone injection on one side of the oral mucosa and a 1-mL PRP injection on the other side every 2 weeks for 3 months. The primary endpoint was the improvement of oral PV lesions in 3 months. Nine out of 11 participants completed the protocol, and 7 (78%) of those 9 patients showed improvement in oral PDAI and/or pain scores at the PRP injection sites. Although PRP resulted in clinical improvement, as in the previous study, there were no significant differences between PRP and intralesional steroid injections (62). Thus, autologous PRP might be used for the treatment of resistant oral erosions in pemphigus patients when intralesional steroid injection is contraindicated.

### Future Potential Clinical Trials in Pemphigus

B cell depletion therapy using an anti-CD20 monoclonal antibody leads to short-term remission in the majority of pemphigus patients; however, we often observe relapsing disease after treatment. Although disease remission is related to the depletion of circulating Dsg3-specific B cells, the expansion of the same pathogenic B cell clone is observed during relapsing disease (63). Therefore, to maintain complete remission in PV, targeted removal of anti-Dsg3 memory B cells is essential. Recently, chimeric antigen receptor (CAR) technology was developed and resulted in novel treatments that led to the prolonged remission of refractory B cell leukemia and lymphoma. Adapted from that strategy, Dsg3 chimeric autoantibody receptor T cell (Dsg3-CAART) therapy has been reported to result in serological and histological improvements in experimental pemphigus mice without detectable off-target toxicity (15). A phase 1 clinical trial of Dsg3-CAART in PV patients is planned to investigate its safety and therapeutic potential.

## Pemphigoid

### Ixekizumab in BP

Th17 cells were first identified by their production of interleukin (IL)-17 (64, 65). A previous study indicated that the transfer of IL-17-producing Th17 cells into healthy SJL/J mice induced experimental autoimmune encephalomyelitis an animal model of MS (66). The prominent role of the Th17 polarization of T cells, as well as that of IL-17, in inflammation and autoimmunity was recognized after this observation. In addition to EAE, the Th17-IL-17 axis is also important in the pathogenesis of RA (67–69). Notably, neutralizing anti-IL-17 antibodies significantly decreased joint inflammation, cartilage destruction, and bone erosion in a collagen-immunized arthritis mouse model. Several recent studies suggest that Th17 cells and their cytokines also play roles in the pathogenesis of AIBD, including BP (70). More specifically, Le Jan et al. reported that IL-17A expression was significantly higher in both serum and lesional skin in BP patients than in normal healthy individuals (71). Furthermore, Chakievska et al. reported that IL-17A -/- mice were protected from the development of experimental BP induced by the transfer of anti-COL17 IgG (72). Taken together, these results indicate that IL-17A is a potential target for the treatment of BP. Ixekizumab is a recombinant fully humanized monoclonal antibody targeting IL-17 and is used for the treatment of psoriasis (73). However, there are neither case reports nor clinical studies reporting the effect of ixekizumab or other IL-17-targeting compounds on BP. To determine the efficacy of ixekizumab for the treatment of BP, an open-label, single-group phase 2 clinical trial is currently ongoing (**NCT03099538**). In this trial, twelve BP patients will receive subcutaneous injections of 160 mg ixekizumab on day 0, and then they will receive 80 mg of ixekizumab every 2 weeks until week 12. The primary endpoint is the median time from the start of treatment to the cessation of blister formation during the 12 weeks of therapy. There is no available report of the results of this trial to date.

### NPB-01 in BP Unresponsive to Corticosteroids

With regard to pemphigus, the efficacy and safety of IVIg in refractory BP patients has been suggested by case reports and case report series (74–76). Based on these observations, several guidelines and consensus statements recommend the use of IVIg for refractory BP (77–79). Recently, Sasaoka et al. reported that IVIg reduced serum IL-6 concentrations, circulating pathogenic IgG levels, and skin inflammation disease activity in an active BP mouse model (80). Kamaguchi et al. reported that an anti-idiotypic antibody in IVIg significantly reduced the BP180 depletion of cultured keratinocytes stimulated with BP pathogenic IgG (81). To evaluate the effectiveness of IVIg in BP, a multicenter, randomized, double-blind, placebo-controlled, phase 3 clinical trial was conducted (**NCT01408550**). In this trial, 56 BP patients were parallelly assigned to receive either IVIg or a placebo. The IVIg group received an intravenous drip infusion of human IgG at 400 mg/kg/day for 5 consecutive days. The primary endpoint was the disease activity score (DAS) of skin lesions on day 15. The results of that study have recently been published (82). The DAS on day 15 was evaluated as the primary endpoint. Although the DAS on day 15 in the IVIg group (19.8 ± 22.2) was 12.5 points lower than that in the placebo group (32.3 ± 31.5), the difference between the groups was not significant (*p* = 0.089). However, a *post hoc* analysis of covariance using the DAS on day 1 as a covariate showed a significant difference between the IVIg and placebo groups (*p* = 0.041). Regarding the safety of IVIg in BP, the incidence of adverse drug reactions was 37.9% (*n* = 11/29) in the IVIg group vs. 18.5% (*n* = 5/27) in the placebo group. No significant difference in the incidence of adverse drug reactions was observed between the IVIg and placebo groups (*p* = 0.143). No patients in either group experienced any severe adverse drug reactions. Therefore, this report suggested that IVIg is a beneficial treatment modality for refractory BP cases treated with a moderate dose of systemic steroids.

### Topical AC-203 in BP

The nucleotide-binding domain, leucine-rich repeat family, pyrin domain-containing 3 (NLRP3) inflammasome regulates the activation of caspase-1 and the release of pro-inflammatory cytokines such as IL-1β and IL-18 in macrophages (83–87). It is an essential innate immune sensor that is activated in response to various damage-associated molecular patterns (83, 84). Previous reports have suggested that dysregulation of the inflammasome attenuates several chronic diseases, including autoimmune diseases such as EAE (88) and systemic lupus erythematosus (89, 90). In addition, polymorphisms in both NLRP3 and caspase-1 recruitment domain-8 genes led to increased IL-1β production and are related to disease susceptibility and severity in RA (91). A recent study demonstrated that the expression levels of NLRP3 and inflammasome components are significantly higher in peripheral blood mononuclear cells from BP patients than in those from healthy donors (92). Furthermore, higher NLRP3 levels were positively correlated with BP disease activity. Although the precise mechanism by which NLRP3 contributes to pathogenesis in BP is still unclear, pharmacological modulation of the inflammasome pathway could be a novel therapeutic strategy in BP. However, in a mouse model of inflammatory EBA, caspase-1/11-deficient mice developed clinical disease manifestations identical to those of the wild-type controls after the injection of autoantibodies against type VII collagen (93).

To evaluate the therapeutic efficacy of caspase-1 inhibition in BP, AC-203, which is a topical ointment formulation of a modulator of the inflammasome and IL-1β pathways, was developed. To investigate the effects of AC-203 on BP, a randomized, open-label phase 2 clinical trial is currently conducted (**NCT03286582**). Forty BP patients received either topical AC-203 or clobetasol 0.05% topical ointment twice a day (time frame is 10 weeks). The primary endpoint is the incidence of adverse effects during the treatment period. No results of this trial are currently available.

### Bertilimumab in BP

While the presence of eosinophils in the dermal infiltrate is one of the histological hallmarks of BP, their contribution to the pathogenesis of BP remains to be determined (94–97). Recent data, however, support the notion that eosinophils promote subepidermal blistering in BP (98). In line with the presence of eosinophils in the dermal infiltrate in BP, elevated expression levels of IL-5, eotaxin, and eosinophil colony-stimulating factor were detected in BP blister fluids (99). Furthermore, matrix metallopeptidase 9 is secreted by eosinophils in BP lesional skin (100), eosinophil cationic protein (ECP) is detected at the dermal-epidermal junction of lesional skin, and serum concentrations of ECP correlate with BP disease activity (101, 102). Based on these findings, eotaxin, which is released upon eosinophil activation (103), has emerged as a therapeutic target for the treatment of BP.

Bertilimumab is a fully human monoclonal antibody targeting eotaxin-1 (104). Therefore, the administration of bertilimumab is expected to impair eosinophil infiltration into the skin in BP. This hypothesis has been recently tested in an open-label, single arm, phase 2 clinical trial (**NCT02226146**). Eleven moderate to severe BP patients received 10 mg/kg of bertilimumab intravenously on days 0, 14, and 28 with approximately 13 weeks of follow-up. The primary endpoint was safety, including the incidence of adverse effects. The results of the study were presented at the 2018 American Academy of Dermatology annual meeting. Of the 11 subjects enrolled, 2 patients withdrew consent and 9 received bertilimumab. Bertilimumab was well tolerated in all 9 subjects, and no drug-associated serious adverse events were reported^4^ Preliminary analyses indicated that the subjects had an 81% decline in their Bullous Pemphigoid Disease Area Index (BPDAI) scores. Based on these results, bertilimumab has been granted fast track designation for the treatment of BP^5^.

### Rituximab in BP

Rituximab has a well-documented efficacy in treating pemphigus (24). However, controlled clinical trials in pemphigoid diseases, including BP, are missing. Several case reports and case report series reported good outcomes of rituximab treatment for BP. Although the efficiency of rituximab for the treatment of BP varied among the studies, the overall CR rates were 60–70% and the relapse rates were approximately 20% in BP patients receiving rituximab therapy (105, 106). To evaluate the efficacy and safety of rituximab for the treatment of BP, an open-label, prospective, phase 3 clinical trial was conducted (**NCT00525616**). Eighteen BP patients received two intravenous injections of 1,000 mg of rituximab at 15-day intervals. The primary endpoint was clinical and biological disease control for up to 2 years. To date, no results of this trial are available.

### Future Potential Clinical Trials in Pemphigoid

Spleen tyrosine kinase (SYK) is a nonreceptor cytoplasmic enzyme that is mainly expressed in hematopoietic cells and plays a pivotal role in regulating cellular responses to extracellular pathogens or antigen-immunoglobulin complexes (107). Because SYK acts downstream of activating Fc gamma receptor (FcγR), it has been considered a candidate drug target for antibody-induced diseases, such as pemphigoid diseases. In fact, a previous study has shown that SYK inhibition led to less activation of neutrophils stimulated with immune complexes and prevented the development of skin lesions in a preclinical EBA mouse model (108). In lesional skin in pemphigoid diseases, both eicosanoid leukotriene B4 (LTB4) and complement factor C5, the precursor of anaphylatoxin C5a, are found; however, their significance in the pathogenesis of pemphigoid diseases still needs to be elucidated. A previous preclinical mouse model of pemphigoid disease indicated a critical role of LTB4 in the recruitment of polymorph nuclear cells to the dermal epidermal junction (109). It is suggested that LTB4 may closely interact with C5a in the regulation of skin inflammation. Thus, inhibiting these two factors individually or in parallel might be effective for the treatment of pemphigoid diseases. These newly identified therapeutic targets should be addressed in future clinical trials.

## Conclusions

The clinical trials discussed here, which include several trials investigating novel therapeutic targets, demonstrate that translational research in pemphigus and pemphigoid is a fast-growing field. We thus expect that several novel treatments will be shortly available for the treatment of pemphigus and pemphigoid patients. Given the high, and thus far unmet, medical need in this field (110), this is highly encouraging and will hopefully improve the quality of life of the affected patients. In addition to the compounds and targets described here, several new targets have been recently identified in preclinical model systems, such as PDE4 or PI3Kδ inhibitors (111, 112). Hence, the preclinical pipeline is well developed and will contribute to the growing number of clinical trials in pemphigus and pemphigoid.

## Author Contributions

KI and RL wrote the manuscript. KB prepared the figure. All authors read, commented on, and approved the final version of the manuscript.

### Conflict of Interest Statement

During the last 3 years, RL has received research funding from Almirall, True North Therapeutics, UCB Pharma, ArgenX, TxCell, Topadur, Incyte and Admirx and fees for consulting or speaking from ArgenX, Immunogenetics, Novartis and Lilly. The remaining authors declare that the research was conducted in the absence of any commercial or financial relationships that could be construed as a potential conflict of interest.

## Footnotes

^1^https://clinicaltrials.gov/

^2^https://www.clinicaltrialsregister.eu/

^3^https://ir.principiabio.com/news-releases/news-release-details/principia-biopharma-reports-positive-prn1008-phase-2-top-line

^4^https://www.immunepharma.com/what-we-do/bertilimumab

^5^https://ir.immunepharma.com/press-releases/detail/168/bertilimumab-granted-fast-track-designation-for-the

## Abbreviations

AIBDs: autoimmune bullous dermatoses
PV: pemphigus vulgaris
PF: pemphigus foliaceus
BP: bullous pemphigoid
EBA: epidermolysis acquisita
p200: anti-laminin-γ1/p200 pemphigoid
PG: pemphigoid gestationis
LPP: lichen planus pemphigoides
LAD: linear IgA bullous dermatosis
DH: dermatitis herpetiformis
RA: rheumatoid arthritis
MS: multiple sclerosis
CR: complete remission
MMF: mycophenolate mofetil
BAFF: B cell activating factor of the tumor necrosis factor family
BAFF-R: B cell activating factor of the tumor necrosis factor family receptor
BCR: B cell receptor
PDAI: Pemphigus Disease Area Index
FcRn: neonatal Fc receptor
IVIg: intravenous immunoglobulin
BTK: Bruton's tyrosine kinase
Tregs: regulatory T cells
GVHD: graft vs. host disease
Dsg: desmoglein
PRP: Platelet-rich plasma
CAR: chimeric antigen receptor
Dsg3-CAART: Dsg3 chimeric autoantibody receptor T cell
IL: interleukin
EAE: experimental autoimmune encephalomyelitis
DAS: disease activity score
NLRP3, nucleotide-binding domain, leucine-rich repeat family: pyrin domain-containing 3
BPDAI: bullous pemphigoid disease area index
ECP: eosinophil cationic protein
SYK: spleen tyrosine kinase
FcγR: Fc gamma receptor
LTB4: leukotriene B4.
