# Supplementary material for: Current Clinical Trials in Pemphigus and Pemphigoid
Source: Front Immunol. 2019 May 3;10:978. doi: 10.3389/fimmu.2019.00978 (PMC6509547; doi:10.3389/fimmu.2019.00978)
Supplement: Supplementary file 1 [file Table_1.DOCX]

**Supplementary Table 1. Clinical trials for pemphigus before 2013**

| NCT number | Disease | Interventions | Target | Allocation | Masking | Phase | Status |
| --- | --- | --- | --- | --- | --- | --- | --- |
| NCT01313923 | PV or PF | Sirolimus | Immune suppression | Single group | None | 1 | Terminated |
| NCT01299857 | PV and PF | Rituximab | CD20 | Single group | None | 3 | Completed |
| NCT00626678 | PV | Azathioprine | Purine synthesis | Randomized | Tripple blind | 2 | Completed |
| NCT00483119 | PV | Intravenous immunogloburin  Cyclophosphamide | FcRn  Immune suppression | Randomized | None | 2 | Terminated |
| NCT00127764 | PV | Adjuvant Oral Glucocorticoid Pulse Therapy | Immune suppression | Randomized | Double blind | 2/3 | Completed |
| NCT00213512 | PV and PF | Rituximab | CD20 | Single group | None | 2/3 | Completed |
| NCT00283712 | PV | Infliximab | TNF-alpha | Randomized | Double blind | 2 | Completed |
| NCT00606749 | PV | KC706 | p38 MAP kinase | Single group | None | 2 | Completed |
| NCT00784589 | PV and PF | Systemic steroid  Rituximab | Immune suppression  CD20 | Randomized | None | 3 | Completed |
| NCT00135720 | PV | Etanercept | TNF-alpha | Randomized | Double blind | 2 | Completed |
| NCT00429533 | PV | Dapsone | Folic acid synthesis | Randomized | Double blind | 2 | Terminated |
| NCT00063752 | PV | PI-0824 | Anergy induction of pathogenic T cells | Single group | None | 1 | Completed |
| NCT00683930 | PV | MMF | IMPDH | Randomized | Double blind | 3 | Completed |
| NCT00656656 | PV or PF | Combination of protein A immunoadsorption, rituximab, dexamethasone plus azathioprine | Pathogenic IgG, CD20,  immune suppression,  purine synthesis | Single group | None | 2 | Completed |
| NCT00010413 | PV or PF | Combination of cyclophosphamide,  plus filgrastim | Immune suppression  Blood cells recovering | Single group | None | 2 | Completed |

PV, pemphigus vulgaris; PF, pemphigus foliaceus; FcRn, neonatal Fc receptor; TNF, tumor necrosis factor; MAP, mitogen-activated protein; IMPDH, inosine 5’-monophosphate dehydrogenase. Terminated: The study has stopped early and will not start again. Participants are no longer being examined or treated. Completed: The study has ended normally, and participants are no longer being examined or treated (that is, the last participant's last visit has occurred).

**Supplementary Table 2. Clinical trials in pemphigoid before 2013**

| NCT number | Disease | Interventions | Target | Allocation | Masking | Phase | Status |
| --- | --- | --- | --- | --- | --- | --- | --- |
| NCT01688882 | BP | QGE031 | IgE | Randomized | Quadruple blind | 2 | Terminated |
| NCT01705795 | BP | Mepolizumab | IL-5 | Randomized | Double blind | 2 | Completed |
| NCT01571895 | BP | DF2156A | CXCR1, CXCR2 | Single group | None | 2 | Terminated |
| NCT00431119 | BP | Azathioprine  MMF | Purine synthesis,  IMPDH | Randomized | None | 2 | Completed |
| NCT00472030 | BP | Omalizumab  Prednisone | IgE | Randomized | None | 4 | Completed |
| NCT00584935 | OCP | Rituximab | CD20 | Single group | None | 1/2 | Completed |
| NCT00286325 | BP | Rituximab | CD20 | Single group | None | 1/2 | Completed |
| NCT00213421 | BP | Dermoval | Immune suppression | NA | NA | NA | Completed |

IL, interleukin; CXCR, C-X-C chemokine receptor; MMF, mycophenolate mofetil; IMPDH, inosine 5’-monophosphate dehydrogenase; OCP, ocular cicatricial pemphigoid; NA, not available. Terminated: The study has stopped early and will not start again. Participants are no longer being examined or treated. Completed: The study has ended normally, and participants are no longer being examined or treated (that is, the last participant's last visit has occurred).
